# The Preparation of Acryloxyl Group Functionalized Siloxane Polymers and the Study of Their Ultra Violet Curing Properties

**DOI:** 10.3390/polym16040465

**Published:** 2024-02-07

**Authors:** Dan Du, Xupeng Chen, Yue Wu, Chuan Wu, Zhirong Qu, Yanjiang Song, Dawei Qin, Qiao Li, Hong Dong

**Affiliations:** 1 College of Material, Chemistry and Chemical Engineering, Key Laboratory of Organosilicon Chemistry and Material Technology, Ministry of Education, Hangzhou Normal University, Hangzhou 311121, China; 2021111017013@stu.hznu.edu (D.D.); 2022112009027@stu.hznu.edu (X.C.); 2022112009058@stu.hznu.edu (Y.W.); chuanwu@hznu.edu.cn (C.W.); 20100123@hznu.edu.cn (Z.Q.); 20080095@hznu.edu.cn (Y.S.); 2 Zhejiang Wynca Chemical Group Co., Ltd., Hangzhou 311600, China; qin_dw@wynca.com; 3 Zhejiang Kaihua Synthetic Material Co., Ltd., Affiliated to Zhejiang Wynca Chemical Group Co., Ltd., Quzhou 324300, China

**Keywords:** radical UV curing, acryloyloxy-functionalized polysiloxane, asymmetric polysiloxane, gelation yield, water contact angle

## Abstract

Polysiloxane with multiple acryloxyl groups at the terminal site of the polymer chain was synthesized by the condensation reaction between hydroxyl-terminated polysiloxane and acryloyl chloride and used to improve the cross-linking density of UV-curable silicone materials initiated from dual acryloxy-terminated symmetric polysiloxane or single acryloxy-terminated asymmetric polysiloxane with the mixture of Irgacure 1173 and Irgacure 184 at a mass ratio of 1:1 as the photoinitiator. The effects of factors such as initiator composition, UV irradiation time, structure, and molecular weight of linear dual acryloxy-terminated or single acryloxy-terminated asymmetric siloxane oligomers on the gelation yield, thermal properties, water absorption, and water contact angle of UV-cured film were investigated. The synthesized cross-linking density modifier can be copolymerized with acryloxy-functionalized linear polysiloxanes under the action of a photoinitiator to increase the cross-link density of UV-cured products effectively. Both linear dual acryloxy-terminated or single acryloxy-terminated asymmetric siloxane oligomers can be copolymerized with cross-link density modifiers within 20 s of UV irradiation. The gelation yields of the UV-cured products obtained from the dual acryloxy-terminated siloxane oligomers were greater than 85%, and their surface water contact angles increased from 72.8° to 95.9° as the molecular weight of the oligomers increased. The gelation yields of UV-cured products obtained from single acryloxy-terminated asymmetric siloxane oligomers were less than 80%, and their thermal stabilities were inferior to those obtained from the dual acryloxy-terminated siloxane oligomers. However, the water contact angles of UV-cured products obtained from these single acryloxy-terminated asymmetric siloxane oligomers were all greater than 90°.

## 1. Introduction

Traditional silicone materials are primarily prepared through thermal curing. The preparation process has problems, such as high energy consumption, long time, and cumbersome operations [1]. With the increasing awareness of environmental protection and the implementation of increasingly stringent environmental regulations in various countries and regions, UV curing technology, with the advantages of low energy consumption, fast curing, and environmental friendliness, has attracted widespread attention from academia and industry [2,3].

Benefitting from the high bond energy, long bond length, and sizeable Si-O-Si bond angle of the Si-O bonds in the molecule, silicone polymer materials have excellent high- and low-temperature resistance, weather resistance, thermal and cold shock resistance, and low surface energy [4,5]. Polysiloxane can absorb energy near the ultraviolet range and is not prone to yellowing or degradation. Therefore, UV-cured polysiloxane materials, such as antifouling materials [6,7,8], hydrophobic materials [9,10,11], and anti-corrosion materials [12], have been widely used.

According to the curing mechanism, UV-cured polysiloxane can be divided into cationic and free radical curing [13]. Cationic UV curing means that the photoinitiator generates superprotonic solid acid or Lewis acid under the energy of ultraviolet light irradiation, forming an intense acidic active center. The styrene-modified silicone materials containing an unsaturated C=C double bond or epoxy group modified silicone materials containing active epoxy group are cross-linked or vulcanized under the action of these acidic active centers. The characteristic of cationic UV curing is that the curing process is not affected by oxygen in the system or environment; that is, there is no so-called “oxygen inhibition” problem. With the extension of UV irradiation time, the cross-linking density of silicone prepolymer molecules gradually increases, and the solvent resistance of the cured product gradually increases. In addition, the volume shrinkage of the polymer before and after cationic UV curing is minor relative to free radical UV curing [14], and the adhesion between the polymer and the substrate after curing is enhanced with the extension of UV irradiation time. Although cationic UV curing is not influenced by oxygen inhibition, cationic UV curing is affected by the steric hindrance effect of polymer active functional groups, which decreases its polymerization speed. Additionally, cationic curing is also inhibited by water [15], alcohols [16], and traces of basic components, such as amines [17]. On the other hand, there are fewer types of characteristic functional groups and polymers suitable for cationic UV curing, resulting in high prices [18,19,20].

A free radical UV photocuring agent is a photoinitiator that decomposes C-C bonds after UV irradiation to generate free radicals and triggers the three reactions of chain initiation, chain transfer, and chain termination of silicone polymers containing acryloyloxy functional groups [21]. Under UV light irradiation, photoinitiators are readily decomposed into free radicals containing unpaired electrons. These free radicals can combine with other compounds at the unpaired electron position or be added to the monomer’s double bond to generate another active free radical. During polymerization, active centers are continuously repositioned at the ends of the growing polymer chain. The polymerization reaction is terminated when a polymer chain free radical reacts with another free radical [22]. Although free radical curing is easily affected by oxygen inhibition and has problems, such as considerable volume shrinkage of the polymer before and after curing and poor adhesion between the polymer and the substrate, the free radical UV curing speed is much faster than the cationic UV curing speed, which made it more efficient, fast, and low cost. Free radical curing has dominated the market for photo-polymerized products [23,24,25].

According to the type of photosensitive group grafted in the polysiloxane molecule, polysiloxanes suitable for UV curing can be divided into thiol-vinyl functionalized polysiloxane [26,27], epoxy-functionalized polysiloxane [28], vinyl ether modified polysiloxane [29], styrene-based polysiloxane, acrylic polysiloxane [30,31], and so on. Thiol-vinyl functionalized polysiloxane can be applied to the surface of polyester film, paper, polyethylene, metal, and other substrates. Its advantage is that the cured film has good elasticity and flexibility [26], and its shortcomings include harshness, poor stability, short shelf life, pungent odor, and high cost [27]. Epoxy-functionalized polysiloxane is mainly used in the field of electronic packaging. Most of these silicone polymers use cationic UV curing methods, requiring photosensitizers and photoinitiators to coordinate during the curing process. Therefore, the requirements for the material’s light transmittance and anti-yellowing properties are relatively high, which poses challenges to the type and dosage of photosensitizers. The vinyl ether polysiloxanes can be used as additives in cationic curable photosensitive compositions to decrease the surface tension of the mixture, acquire better wetting and adhesion to aluminum, and compete with conventional fluorinated surfactants [29]. After introducing the styrene-based functional group into the polysiloxane molecule, the cross-linked silicone material has higher hardness, excellent mechanical properties, and lower material cost. Acrylated polysiloxane is mainly used in UV radical curing processes, and UV light-cured acryloxy-functionalized polysiloxane can produce coatings with excellent thermal stability. Introducing polysiloxane segments into the coating can effectively reduce the surface energy of the coating, which effectively promotes the application of UV-cured polysiloxane in the coating field. Therefore, the research and development of UV light-cured acryloxy-functionalized polysiloxane materials has attracted much interest in recent research on photocurable materials [30,31].

Although water-based polyurethane acrylate resin can also be UV-cured to obtain environmentally friendly water-based coatings, it is inevitable to introduce toxic isocyanate functional groups during the polymer synthesis process, resulting in certain safety risks in the synthesis of this type of polymer. Compared with polyurethane acrylate resin, acrylate functionalized silicone polymers are easy to synthesize [32,33], require lower raw material costs, and have broader application fields. They are currently mainstream commercial products and usually follow the free radical UV light curing mechanism. The position and content of the acryloxy functional group in the polysiloxane molecule are the main factors affecting the performance of photocured products and determining the application fields of UV photocurable silicone coating products.

Li and his colleagues used pentaerythritol triacrylate (PETA) as a raw material. They introduced multiple acryloxy functional groups at both ends of the silicone polymer through the hydrosilylation reaction between the C=C double bond and the Si-H bond to prepare UV-curable silicone materials with enhanced cross-linking density, transparency, oil resistance, and high-temperature resistance. However, since the four acryloyloxy groups contained in the PETA can all undergo hydrosilylation with the Si-H bond, the structure of the hydrosilylation product is uncertain, and there are numerous by-products [34].

Apart from the hydrosilylation reaction, acryloyloxy-functionalized polysiloxanes can also be prepared through the condensation reaction between acrylic acid and hydroxyl-functionalized polysiloxanes. However, since the condensation reaction is reversible, the reaction yield is low, and the product composition is relatively complex.

In order to overcome the drawback of the low yield of a condensation reaction between acrylic acid and hydroxyl-functionalized polysiloxane in the construction of acryloyloxy-functionalized polysiloxane and overcome the shortcomings of uncertain product structure in hydrosilylation between PETA and Si-H bonds, the condensation reaction between acryloyl chloride (AC) and hydroxyl-functionalized polysiloxane was utilized in this manuscript to prepare acryloxy-functionalized polysiloxane. Organic amines were applied as the acid-binding agents to promote the reaction. Thus, an oligomer with a high content of acryloyloxy functional groups was obtained. To further increase the content of UV-curable acryloxy functional groups in the siloxane molecules and improve the gelation yield of the cured product, a telechelic oligomer containing multiple acryloxy functional groups at both ends was also designed and synthesized. The influence of various factors, such as the structure and molecular weight of the oligomer, the molecular structure of the telechelic oligomer, and the composition and dosage of the photoinitiator on the structure and properties of the UV-cured product were investigated, and these studies might guide the research and development of UV light-curable silicone materials.

## 2. Materials and Methods

### 2.1. Raw Materials

*α*,*ω*-bis[(dimethyl)(γ-hydroxyethyloxypropyl)]-terminated polydimethylsiloxane (DHEP-PDMS) with *M*_NMR_ = 1050, 1910, 3020, and 4010 g/mol and *α*,*ω*-bis(dimethylsilyl)-terminated polydimethylsiloxane (DDMS-PDMS) with *M*_NMR_ = 5680 g/mol determined by ^1^H NMR spectra were purchased from Jiaxing United Chemical Co., Ltd. (Jiaxing, China). *α*-trimethylsilyl-*ω*-dimethyl-terminated polydimethylsiloxane (MDMS-PDMS) oligomers with *M*_NMR_ = 550, 960, 1440, and 2220 g/mol determined by ^1^H NMR spectra were purchased from Zhejiang Equation New Material Co. Ltd. (Quzhou, China). Acryloyl chloride (AC), allyl hydroxyethyl ether (AHE), 2-Hydroxy-2-Methyl-Phenyl-Propane-1-one (Irgacure 1173, HMPP), and 1-Hydroxy-Cyclohexyl-Phenyl-Ketone (Irgacure 184, HCPK), all AR grades, were purchased from Jiuding Chemical (Shanghai, China) Technology Co., Ltd. (Shanghai, China). Trimethylolpropane allyl ether (TMPAE) was purchased from Sigma-Aldrich Co. LLC (Burlington, MA, USA). Dichloromethane (DCM), ethyl acetate (EA), triethylamine (TEA), NaHCO_3_, and NaCl, all AR grades, were purchased from Sinopharm Chemical Reagent Co., Ltd. (Shanghai, China). Na_2_SO_4_, AR grade, was purchased from Shanghai Lingfeng Chemical reagents Limited (Shanghai, China). 

### 2.2. Characterization and Instruments

The dynamic viscosity of each liquid sample at 25 °C was measured using a rotational viscometer (Brookfield DV2TRVTJ0, Brookfield, WI, USA). Gel permeation chromatography (GPC) was measured on a PL-GPC 50 (Agilent, Santa Clara, CA, USA) with toluene as the mobile phase, polydimethylsiloxane with a known molecular weight (490, 6200, 15,000, 34,100, 64,800, 95,800, 219,400, 384,500, and 610,000 g/mol, American Polymer Standards Corporation, Mentor, OH, USA) as the standard, and a Styragel HT2 as the separation column. The weight average molecular weight (*M*w), number average molecular weight (*M*n), and polydispersity index (PDI) of the polymer were obtained on the basis of GPC test results with a toluene flow rate of 1.0 mL/min and a column oven temperature of 35 °C.

The ^1^H NMR spectrum of each sample was measured on an AVANCE AV 400 NMR spectrometer (Bruker, Bremen, Germany). During the test, approximately 10 mg of the sample was dissolved in approximately 0.6 mL of deuterated chloroform (CDCl_3_) containing no internal standard, and the relaxation time D_1_ was set to 5 s. The ^13^C NMR spectrum of each polymer was measured on a solid–liquid dual-purpose Avance AV III 500 NMR spectrometer (Bruker, Germany). During the test, approximately 100 mg of the sample was dissolved in approximately 0.6 mL of CDCl_3_ without internal standard, and the relaxation time D_1_ was 3 s.

The FT-IR spectra of raw materials, intermediate products, and final products were recorded on a Bruker Alpha-T Fourier-transform infrared spectrometer when the KBr tableting method was used, and the test range was between 4000 and 400 cm^−1^ with a resolution of 4 cm^−1^. A Discovery TGA (TA Instruments Company, New Castle, DE, USA) was used to measure the relationship between the mass of UV-cured products and the temperature changes at a nitrogen flow rate of 15 mL/min. The test range was between 40 and 800 °C, and the heating rate was 20 °C/min. A KRUSS DSA 30 water contact angle measuring instrument (KRUSS Company, Hamburg, Germany) was used to test the water contact angle of the smooth surface of each UV-cured sample. The water contact angle value of each sample was measured three times at different positions, and the average value was taken. A differential scanning calorimeter (DSC, Netzsch DSC 214, Selb, Bavaria, Germany) was used to test the structural stability of UV-cured products at different temperatures in a nitrogen atmosphere. During the test, the temperature was first cooled from room temperature to −140 °C, then raised from −140 °C to 150 °C, and finally dropped to room temperature. The heating and cooling rates were both set to 10 K/min. The UV-cured films of acryloxy group functionalized polysiloxane oligomers were conducted on a drawer-type UV curing machine with a Hg-UV lamp power of 80~100 mW and an electric power of 2.2 kW (UU-10-20/1, Kunshan Richang Huaxin Electronic Materials Co., Ltd. (Suzhou, China) in a nitrogen atmosphere. 

The gelation yield, water absorption degree, and cross-linking density of UV-cured films were tested according to the literature methods [35,36]. During the gelation yield measurement, a certain amount of UV-cured film (*m*_1_) was added to a dry glass bottle. Toluene with a sufficient amount was then added to immerse the sample completely. After that, the glass bottle was sealed with PE film and stood at room temperature for 24 h. After the toluene solution was decanted from the bottle, the glass bottle was placed in the oven and dried at 100 °C for 1 h to completely evaporate the remaining toluene. After cooling to room temperature, the net weight (*m*_2_) of the remaining sample was weighed. The ratio of *m*_2_ to *m*_1_ is the gelation yield of the UV-cured film sample. During the water absorption test, the UV-cured sample was first dried in an oven with a constant temperature of 80 °C for 1 h to ensure that the surface of the sample did not contain moisture. A certain amount of dried sample (*m*_a1_) was weighed and soaked in deionized water for 24 h. The sample was then taken out of the water solution. After the water droplets on the surface of the sample were absorbed with clean filter paper, the sample was weighed and marked as *m*_a2_. When the sample was placed in an oven with a constant temperature of 80 °C for 1 h, the mass of the sample was weighed with a value of *m*_a3_ after the sample temperature was dropped to room temperature. The value of the water absorption was then calculated as (*m*_a2_ − *m*_a3_)/*m*_a1_ × 100%. The same UV-cured sample was tested three times, and its average value was reported as the water absorption of this sample. 

The cross-linking density was determined on the basis of the equilibrium swelling theory introduced by Flory and Rehner. At room temperature (25 °C), approximately 0.1 g of UV-cured film was placed into a sealed glass bottle, and some anhydrous toluene was added to immerse it completely. After the sample was immersed for 4 days, it was taken out from the toluene solution and weighed after the toluene on the surface of the sample was wiped off with the filter paper. Then, continue to soak the sample in the toluene solution, take out the sample every 3 h, wipe it clean, and weigh it. When the mass of the sample no longer changes, the swelling equilibrium is considered to have been reached. The value of *M*_c_ and the cross-linking density *v*_e_ of the sample can be calculated according to Equations (1) and (2)

(1) $$v_{e}=\frac{\rho }{Mc}=-\frac{ln\left(1-\varphi \right)+\varphi +x_{1}\varphi^{2}}{V_{0}\varphi^{\frac{1}{3}}}$$

(2) $$\varphi =\frac{\frac{W_{0}}{\rho }}{\frac{\left(W_{s}-W_{0}\right)}{\rho_{1}}+\frac{W_{0}}{\rho }}$$

where *M*_c_ represents the average molecular weight between two adjacent cross-linking points in the cured sample (g·mol^−1^), *ρ* is the density of the sample (g·cm^−3^), and *ρ*_1_ is the density of toluene (g·cm^−3^). *V*_0_ is the molar volume of toluene, and its value is 106.54 × 10^−3^ L·mol^−1^. *φ* is the volume fraction of PDMS in the swollen sample, which is also the reciprocal of the equilibrium swelling volume. *x*_1_ is the interaction parameter between the polymer and the solvent, and its value is 0.465. *W*_0_ is the initial mass of the sample, and *W*_s_ is the mass of the sample after the swelling equilibrium was reached.

### 2.3. Preparation of DAEP-PDMS, MAEP-PDMS, and DBABP-PDMS Oligomers

#### 2.3.1. *α*,*ω*-Bis(dimethyl-(γ-acryloyloxyethyloxypropyl))-terminated Polydimethylsiloxane (DAEP-PDMS)

**Scheme 1:**
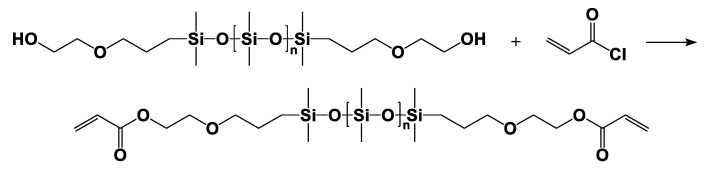
Preparation of the DAEP-PDMS oligomer.

Taking the DAEP-PDMS-1 oligomer in Table 1 as an example, the synthesis of the DAEP-PDMS oligomer using DHEP-PDMS and AC as raw materials was presented in Scheme 1. 

In a 250 mL four-necked flask with a PTFE stirrer, 30 g DCM, 30 g (0.028 mol) DHEP-PDMS with *M*n = 1050 g/mol, and 5.79 g (0.06 mol) TEA were added. The temperature of the mixed materials was cooled to 0 °C in an ice bath. A total of 5.18 g (0.06 mol) AC was then added to the reaction mixture by dropwise addition. The dripping rate of AC was controlled to hold the temperature of the material not higher than 10 °C. After the addition of AC was completed, the mixture was transferred to an oil bath, and the reaction was refluxed at room temperature for 3 h. After the reaction, the salt was removed by suction filtration, and DCM was distilled under reduced pressure at 30 °C/−101 kPa. The remaining liquid in the kettle was washed repeatedly with EA and saturated NaHCO_3_ solution. The oil layer was collected and dried with anhydrous Na_2_SO_4_ for 6 h, and then the low boiling liquid was removed at 70 °C/−101 kPa to obtain 26.28 g of a light-yellow transparent liquid. DAEP-PDMS oligomers with different molecular weights were prepared according to the method described above when DHEP-PDMS oligomers with different molecular weights were used, and the results are listed in Table 1.

#### 2.3.2. α-Trimethylsilyl-ω-dimethyl(γ-acryloyloxyethyloxypropyl)siloxy-terminated Polydimethylsiloxane (MAEP-PDMS)

##### α-Trimethylsilyl-ω-dimethyl(γ-hydroxyethyloxypropyl)siloxy-terminated Polydimethylsiloxane (MAMP-PDMS)

**Scheme 2:**
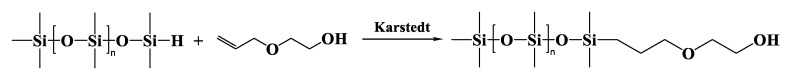
Synthesis of the MAMP-PDMS oligomer by a hydrosilylation reaction.

The preparation method of the MAMP-PDMS oligomer was shown in Scheme 2, and it was synthesized by a hydrosilylation reaction between MDMS-PDMS and AHE. Taking the MAMP-PDMS-1 oligomer as an example, 3.73 g (0.037 mol) of AHE and 0.24 g of Karstedt’s catalyst with effective Pt content of 0.1 wt% (10 ppm) were added to a 100 mL three-necked flask equipped with a thermometer, a condenser tube, a stirrer, and a rubber stopper and purged with nitrogen. The mixture was heated to 70 °C and activated at this temperature for 30 min. When the activation was performed, 20.00 g (0.037 mol) of MDMS-PDMS with *M*_NMR_ = 1050 g/mol was added to the three-necked flask over 2 h, and then the reaction lasted for 5 h. After the reaction was completed, the flow of nitrogen gas was stopped. During the cooling process, the crude reaction product was exposed to air to deactivate the platinum catalyst. After distilling the low boiling substances under reduced pressure at 110 °C and −100.8 kPa, 21.58 g of a transparent light yellow liquid product was obtained. When MDMS-PDMS with other molecular weights was used, MAMP-PDMS oligomers with different molecular weights were prepared using the same method, and the results are shown in Table 2.

##### MAEP-PDMS

**Scheme 3:**
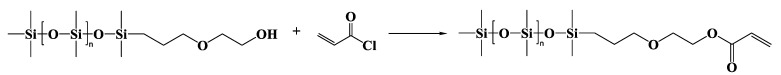
Synthesis of the MAEP-PDMS oligomer by a condensation reaction.

According to the scheme shown in Scheme 3, MAEP-PDMS was prepared by a condensation reaction between MAMP-PDMS and AC. Taking the MAEP-PDMS-1 oligomer as an example, the four-necked flask equipped with a thermometer, condenser tube, and constant pressure dropping funnel, stirrer, and rubber stopper was ventilated three times. After that, the remaining air in the reactor was driven away by nitrogen gas. Subsequently, 15.00 g (*M*_NMR_ = 650 g/mol, 0.023 mol) of the MAMP-PDMS-1 oligomer, 2.34 g (0.023 mol) of TEA, and 30.00 g (0.023 mol) of DCM were added. Stir and reduce the temperature of the mixture to 0 °C with an ice bath. After that, 2.09 g of AC was slowly dropped into the reactor, and the dripping rate was controlled so that the system temperature did not exceed 10 °C. When AC was completely added, the reaction mixture was heated to 25 °C in an oil bath and maintained at this temperature for 6 h. After the reaction was completed, the salt was removed by filtration, and the solvent was removed from the filtrate under reduced pressure at 30 °C. The concentrated solution was transferred to a separatory funnel and washed three times with EA and NaHCO_3_ saturated solution to obtain a neutral upper layer solution. The solvent and low boiling substance contained in the upper layer solution were distilled at 60 °C under a reduced pressure of −100.8 kPa to obtain 14.55 g of a slightly yellow transparent solution. When MAMP-PDMS-1 was substituted by precursors with other molecular weights, MAEP-PDMS oligomers with different molecular weights were obtained using the same method, and the results are listed in Table 3. 

#### 2.3.3. *α*,*ω*-Bis[dimethyl-[3-[2,2-bis(acryloyloxy)butoxy]propyl]silyl]-terminated Polydimethylsiloxane (DBABP-PDMS)

##### *α*,*ω*-Bis[dimethyl-[3-[2,2-bis(hydroxymethyl)butoxy]propyl]silyl]-terminated Polydimethylsiloxane (DBHBP-PDMS)

**Scheme 4:**
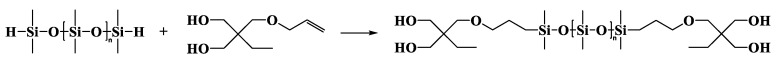
Preparation of the DBHBP-PDMS oligomer.

DBHBP-PDMS was prepared by a hydrosilylation reaction between DDMS-PDMS with an *M*_NMR_ of 5680 g/mol and TMPAE, as shown in Scheme 4. 

In a 100 mL three-necked flask equipped with a thermometer, condenser tube, stirrer, and rubber stopper, and purged with nitrogen, 2.09 g (0.012 mol) of TMPAE and 0.16 g of Karstedt’t catalyst with 0.1 wt% Pt were added under agitation, heated to 70 °C, and activated for 30 min.

After activation, 30.00 g (0.006 mol) of DDMS-PDMS was added to the reactor over 3 h. After the addition of DDMS-PDMS was completed, the reaction lasted for 3 h at this temperature. After the reaction, the nitrogen was turned off; the reaction mixture was exposed to air, cooled to room temperature naturally, and the platinum catalyst was deactivated. The reaction mixture was distilled at 110 °C and a reduced pressure of −100.8 kPa. After the low boiling substance was removed, 29.68 g of transparent colorless liquid was obtained.

##### DBABP-PDMS

**Scheme 5:**
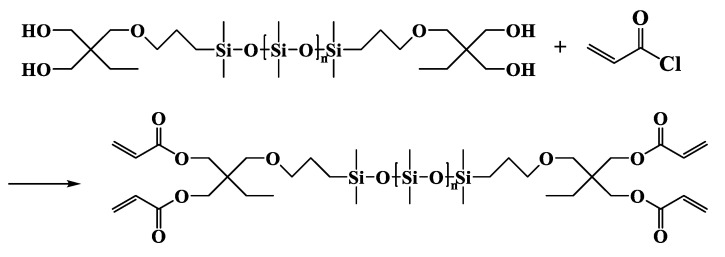
Synthetic route of the DBABP-PDMS oligomer.

As shown in Scheme 5, the preparation of the DBABP-PDMS oligomer was similar to the preparation of the MAEP-PDMS oligomer described in Section 2.3.2. In a 250 mL three-necked flask filled with 60 mL of DCM, 27 g (4.47 × 10^−3^ mol) of the prepared DBHBP-PDMS oligomer was mixed with 1.59 g (0.018 mol) of AC and 1.82 g (0.018 mol) of TEA. After reacting for 3 h, 22.83 g of product was obtained through the same post-treatment process. When using DBHBP-PDMS as the key component, the yield of the reaction was 79.85%.

### 2.4. Preparation of UV-Cured Films

The DAEP-PDMS oligomer was evenly mixed with the telechelic oligomer DBABP-PDMS and the photoinitiator at a mass ratio of 70:30:2. The mixture with a mass of about 1 g was poured into a PTFE mold with a length, width, and thickness of 30 mm, 30 mm, and 6 mm, respectively. The mold was placed in a vacuum oven to remove residual bubbles in the sample and reduce the adverse effects of oxygen inhibition caused by the dissolved oxygen. Then, the degassed mold was placed in a drawer-type UV curing machine and purged with nitrogen gas. The distance between the light source and the sample was about 10 cm. The samples were UV-cured under the set curing time. After the curing was completed, the cured film was taken out, put into a sealed bag, and stored in a dry and dark environment. By changing parameters, such as the composition and dosage of the oligomers and the dosage of the photoinitiator, a series of UV-cured polysiloxane films with different compositions and structures can be obtained under the same UV curing conditions. 

## 3. Results and Discussion

### 3.1. Characterization of the Polymer Structure

#### 3.1.1. GPC Curves

**Figure 1:**
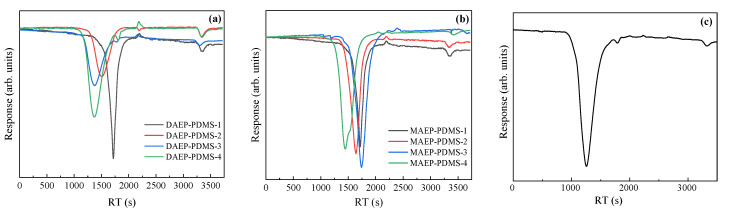
GPC curve of oligomers ((**a**) DAEP-PDMS; (**b**) MAEP-PDMS; (**c**) DBABP-PDMS).

The GPC curves of the prepared DAEP-PDMS, MAEP-PDMS, and DBABP-PDMS oligomers are shown in Figure 1. The GPC curve of each polymer sample showed the typical normal distribution characteristics of high molecular polymers. There was only one peak in the spectrum, which confirmed that the sample prepared was a single-component polymer. It can be observed in Figure 1b that the GPC curve exhibited a non-normal distribution shape, which was due to the low conversion rate of the MAEP-PDMS-4 oligomer.

#### 3.1.2. FT-IR Spectra

##### DAEP-PDMS Oligomer

**Figure 2:**
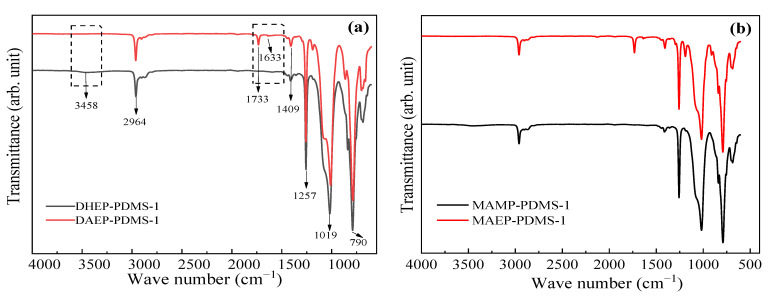
FT-IR spectra of the oligomers of DAEP-PDMS-1 and MAEP-PDMS-1: ((**a**) DHEP-PDMS-1 and DAEP-PDMS-1 oligomers; (**b**) MAMP-PDMS-1 and MAEP-PDMS-1 oligomers).

Taking the DAEP-PDMS-1 oligomer as an example, the FT-IR spectra of DHEP-PDMS-1 and DAEP-PDMS-1 oligomers are shown in Figure 2a. Peaks at 1257 cm^−1^ and 790 cm^−1^ were the stretching vibration peaks of Si-CH_3_, and a peak at 1019 cm^−1^ was the stretching vibration peak of Si-O-Si. A peak at 1409 cm^−1^ was the shearing of the terminal methylene group of the vinyl group, and a vibration absorption peak attributed to the stretching vibration of the C-H bond was observed at 2964 cm^−1^. It can be seen in Figure 2a that when the reaction was completed, the -OH stretching vibration peak resulting from the DHEP-PDMS oligomer located at 3458 cm^−1^ disappeared. At the same time, the C=O stretching vibration peak at 1733 cm^−1^ and the C=C stretching vibration peak at 1633 cm^−1^ appeared in the FT-IR spectrum of the DAEP-PDMS oligomer. These results indicated that the hydroxyl groups in the DHEP-PDMS oligomer underwent a condensation reaction with AC, and the DAEP-PDMS oligomer was successfully prepared.

##### MAEP-PDMS Oligomer

The FT-IR spectra of the MAMP-PDMS-1 and MAEP-PDMS-1 oligomers are shown in Figure 2b. Similar to Figure 2a, the stretching vibration peak attributed to Si-CH_3_, the stretching vibration peak of Si-O-Si, and the shearing peak of the terminal methylene group attributed to the vinyl group, and the stretching vibration peak of the C-H bond could be clearly observed. In Figure 2b, it can be found that the -OH stretching vibration peak in the MAMP-PDMS-1 oligomer disappeared, while the C=O stretching vibration peak and C=C stretching vibration peak generated in the MAEP-PDMS-1 oligomer, indicating that the MAEP-PDMS-1 oligomer was successfully prepared through a condensation reaction between the hydroxyl groups in the MAMP-PDMS-1 oligomer and AC.

#### 3.1.3. NMR Spectra

(A) ^1^H NMR Spectra

DHEP-PDMS and DAEP-PDMS Oligomers

**Figure 3:**
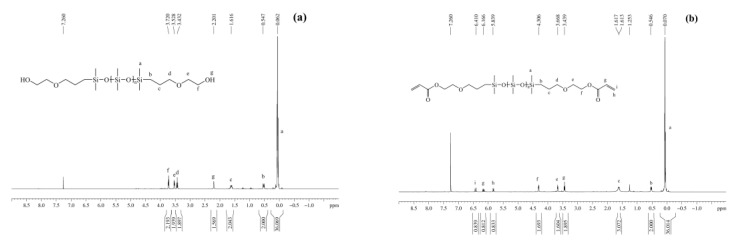
^1^H NMR spectra of the oligomers of DHEP-PDMS-1 (**a**) and DAEP-PDMS-1 (**b**).

Taking the DHEP-PDMS-1 and DAEP-PDMS-1 oligomers (DAEP-PDMS-1 in Table 1) as examples, their ^1^H NMR spectra are shown in Figure 3a,b. The peak near *δ* = 0.0 ppm was the chemical shift of the proton in -Si-CH_3_ groups that attached to the side chain or was located at the terminal site of the DHEP-PDMS and DAEP-PDMS oligomers. The peak near *δ* = 0.5 ppm was the chemical shift of the proton in the methylene group that connected to the Si atom in the -Si-C***H***_2_-CH_2_-CH_2_-O chain segment in the DHEP-PDMS and DAEP-PDMS oligomers. The peak at *δ* = 3.4 ppm was the chemical shift of the proton in the methylene group connected to the O atom in the Si-CH_2_-CH_2_-C***H***_2_-O- chain segment, and *δ* = 3.6 ppm was the chemical shift of the proton marked in the segment of Si-CH_2_-CH_2_-CH_2_-O-C***H***_2_-*. The peak near *δ* = 4.3 ppm was the chemical shift of the proton marked in the segment of Si-CH_2_-CH_2_-CH_2_-O-CH_2_-C***H***_2_-O-*, and the peaks between *δ* = 5.8~6.4 ppm were attributed to the chemical shift of the proton in -C***H***=C***H***_2_ in the acryloxyl group. The peak at *δ* = 7.26 ppm was the chemical shift of the proton in CDCl_3_. 

It can be seen in Figure 3a,b that the chemical shifts of the protons with peaks labeled as a~e did not change before and after the reaction. However, the peak labeled as f (corresponding to the proton in the -C***H***_2_ group) changed from *δ* = 3.7 ppm before the reaction (Figure 3a) to *δ* = 4.3 ppm after the reaction (Figure 3b). If the number of protons at the -Si-C***H***_2_- position was defined as 2, when the reaction was completed, the integration area of the protons labeled in the functional group Si-CH_2_-CH_2_-CH_2_-O-CH_2_-C***H***_2_-O in Figure 3b should be equal to 2. Therefore, the reaction degree of the hydrosilylation could be judged by comparing the integration peak area at *δ* = 4.3 ppm in the ^1^H NMR spectrum of the hydrosilylation product. When the integrated area was divided by 2, the reaction degree of the hydrosilylation was obtained and shown in column 9 of Table 1. The ^1^H NMR spectra of other DAEP-PDMS oligomers were presented in Figures S1–S3 in the Supporting Information.

MAMP-PDMS and MAEP-PDMS Oligomers

**Figure 4:**
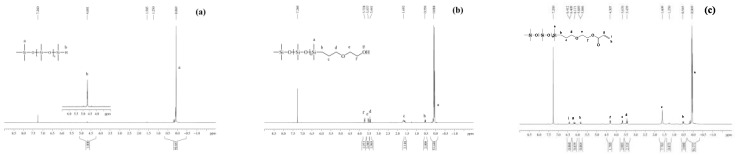
^1^H NMR spectra of the oligomers of MDMS-PDMS-1 (**a**), MAMP-PDMS-1 (**b**), and MAEP-PDMS-1 (**c**).

Taking the MAEP-PDMS-1 oligomer as an example, the ^1^H NMR spectra of the intermediate MAMP-PDMS-1 and its precursor MDMS-PDMS-1 for the synthesis of the MAEP-PDMS-1 oligomer are shown in Figure 4. It can be seen in Figure 4a,b that the peak at *δ* = 4.7 ppm in Figure 4a, the proton signal belonging to Si-H disappeared after the hydrosilylation reaction between MDMS-PDMS and AHE. At the same time, proton peaks attributed to hydroxyl groups and methylene groups appeared in Figure 4b, and the ratios among these integration areas were consistent with the structural formula of MAMP-PDMS, indicating that the condensation of MDMS-PDMS and AHE successfully prepared MAMP-PDMS. It can be observed in Figure 4b,c that after the reaction of MAMP-PDMS with AC, the shape of the proton peak attributed to the hydroxyl group at *δ* = 1.7 ppm changed from a small hill to a sharp peak. Since the protons attributed to the hydroxyl group overlapped with the protons from the moisture in the CDCl_3_ at this position, it was difficult to conclude whether MAEP-PDMS had been successfully prepared through the change in chemical shift at this position.

It can also be observed in Figure 4b,c that signals attributed to the protons attached to C=C appeared in the range of *δ* = 5.7~6.7 ppm in Figure 4c, and the area of each peak was almost identical. At the same time, a proton signal appeared at *δ* = 4.31 ppm in Figure 4c, which was labeled as f and attributed to the proton on the methylene group that connected to the terminal acrylate group. It shifted from *δ* = 3.75 ppm in Figure 4b to *δ* = 4.31 ppm due to the chemical environment of adjacent hydroxyl groups having been replaced by acrylate groups. It could be concluded that the condensation reaction between MAMP-PDMS and AC had successfully produced the MAEP-PDMS oligomer. If the peak area of the proton at *δ* = 0.5 ppm (labeled as b in both Figure 4b,c) was defined as 2, the peak area of the proton labeled as f and appeared at *δ* = 4.31 ppm should be 2. However, the integration area of this signal was 1.789. Therefore, the conversion of the functional group in the synthesis of MAEP-PDMS by the condensation reaction of MAMP-PDMS and AC was calculated as *α* = 1.789/2 × 100% = 89.5%. The ^1^H NMR spectra of other MAEP-PDMS oligomers were presented in Figures S4–S6 in the Supporting Information.

DBHBP-PDMS and DBABP-PDMS

**Figure 5:**
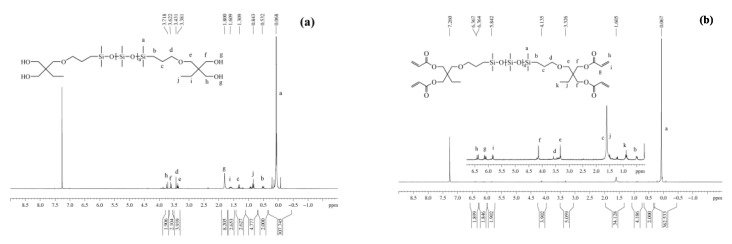
^1^H NMR spectra of the oligomers of DBHBP-PDMS (**a**) and DBABP-PDMS (**b**).

The ^1^H NMR spectra of DBHBP-PDMS and DBABP-PDMS are shown in Figure 5a,b. In Figure 5a, the protons attributed to Si-C***H***_3_ were located at *δ* = 0.00 ppm, and the protons attributed to Si-C***H***_2_-CH_2_ were at *δ* = 0.53 ppm. The protons attributed to -(CH_2_)_3_C-CH_2_-C***H***_3_, Si-C***H***_2_-CH_2_-CH_2_-, and -(CH_2_)_3_C-C***H***_2_-CH_3_ appeared at *δ* = 0.84, 1.31, and 1.61 ppm, respectively. Peaks at *δ* = 1.80 ppm were attributed to the proton in the –OH group or residual trace water in CDCl_3_. The protons marked as e and d in the chain Si-CH_2_-CH_2_-C***H***_2_-O-C***H***_2_-O appeared at about *δ* = 3.43 ppm. Peaks at *δ* = 3.62 ppm and *δ* = 3.72 ppm were the labeled protons in segment -(CH_2_)_2_C-(C***H***_2_-OH)_2_.

Some new proton signals appeared at about *δ* = 5.84~6.37 ppm in Figure 5b, and these protons were attributed to marked protons in the O-C=O-C***H***=C***H***_2_ segment. At the same time, the peak position of the labeled proton in the -(CH_2_)_2_C-(C***H***_2_-OH)_2_ segment also shifted to *δ* = 4.14 ppm. The proton signals labeled as c and j, as well as the proton signals attributed to traces of water remaining in the CDCl_3_, overlapped at *δ* = 1.61 ppm.

The degree of grafting of the acryloyloxy functional group could be calculated from the integrated area at the f position. If the integration area of protons in Si-C***H***_2_- was defined as 2, then the integration area of protons at position f should be 4. However, the integrated area obtained from the ^1^H spectrum was 3.90; the reaction degree was then calculated as α = 3.90/4 × 100% = 97.5%.

(B) ^13^C NMR spectra

**Figure 6:**
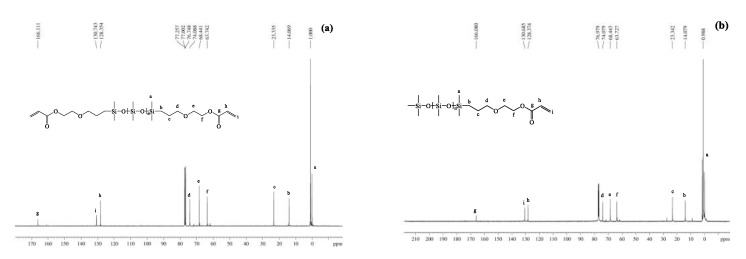
^13^C NMR spectra of the oligomers of DAEP-PDMS (**a**) and MAEP-PDMS (**b**).

The ^13^C NMR spectra of the synthesized DAEP-PDMS and MAEP-PDMS oligomers are shown in Figure 6a,b. Since both DAEP-PDMS and MAEP-PDMS oligomers contained the same functional groups, their ^13^C NMR spectra were almost the same. The peak at *δ* = 1.0 ppm was the chemical shift of the C atom in the side methyl group (-Si-***C***H_3_) in the dimethylsiloxane segment. In the -Si-CH_2_-CH_2_-CH_2_-O- chain segment, the chemical shift of the methylene C atom directly connected to the Si atom, the chemical shift of the methylene C atom adjacent to the -Si-CH_2_- chain segment, and the chemical shifts of the methylene C atom connected to the oxygen atom were at *δ* = 14.1, 23.34, and 77.00 ppm, respectively. In the polymer end group, the chemical shift of the methylene C atom in the *O***C***H_2_CH_2_- chain segment connected to the -Si-CH_2_-CH_2_-CH_2_* chain segment was around *δ* = 68.4 ppm, and the chemical shift of the C atom in the methylene group connected to the acryloyloxy group (-***C***H_2_-OC(=O)-CH=CH_2_) was around at *δ* = 63.74 ppm. In the terminal acryloxy functional group, the chemical shift of the carbonyl C atom was located at *δ* = 166.1 ppm, the chemical shift of the vinyl C atom adjacent to the carbonyl C atom was located at *δ* = 128.35 ppm, and the other C atom in the vinyl group far away from the carbonyl group was located at *δ* = 130.74 ppm.

The ^1^H NMR and ^13^C NMR spectra indicated that the DAEP-PDMS, MAEP-PDMS, and DBABP-PDMS oligomers were successfully prepared through the condensation reaction between AC and terminal hydroxyl groups in the oligomers. 

### 3.2. UV Curing Products

#### 3.2.1. Effect of the Photoinitiator Ratio on Curing Performance

HMPP, with a commercial name of Irgacure 1173, and HCPK, with a commercial name of Irgacure 184, were commonly used photoinitiators for UV-free radical curing. Without the addition of the cross-linking density promotor DBABP-PDMS prepared in Section 2.3.3, a mixture of HMPP and HCPK blended at a mass ratio of 1:1 was added in an amount of 2 wt% of the mass of DAEP-PDMS-1 in Table 1 and irradiated for 20 s in a drawer-type UV curing machine. The gelation yield obtained for this UV-cured film, according to the method described in Section 2.2, was 85.1%. 

In order to investigate the influence of the composition of the photoinitiator on the photocuring process of acryloyloxy-functionalized siloxane oligomers and research the structure and properties of the cured products, DAEP-PDMS-1 in Table 1 or MAEP-PDMS-1 in Table 3 was mixed with the cross-linking density promotor DBABP-PDMS at a mass ratio of 70:30, together with 2 wt% photoinitiators. After the mixture was evenly blended, it was put into a UV light curing machine. A UV-cured film was prepared according to the method described in Section 2.4. A series of UV-cured films were prepared when the ratio of photoinitiator HMPP and HCPK changed while other components remained unchanged. The gelation yield of each UV curable film could be measured according to the method described in Section 2.2, and the results are summarized in Table 4. 

It can be seen in Table 4 that compared with using HMPP alone as the photoinitiator, the gelation yields of the UV-cured film prepared with the mixture of HMPP and HCPK at different mass ratios were slightly improved. When the mass ratio of HMPP to HCPK was 4:1, the gelation yield of the UV light-cured film was the highest, reaching 89.8%. However, compared with using HMPP alone as a photoinitiator, the average gelation yield of films prepared using a mixture of HMPP and HCPK as the photoinitiator only increased by 0.4%. On the other hand, when HMPP and HCPK were mixed in different proportions, the gelation yields of the UV-cured films obtained were almost unchanged, which indicated that the synthesized DAEP-PDMS oligomers had the characteristics of rapid cross-linking, and thus the changes in photoinitiator composition had little influence on the gelation yields of the UV-cured films. Therefore, when considering the curing performance and the price of the photocuring agent, the mass ratio between HMPP and HCPK was recommended to be 1:1. Comparing the gelation yield results listed in Table 4 with the gelation yield when DBABP-PDMS was absent, it could be found that the addition of the DBABP-PDMS had greatly enhanced the cross-linking density of the cured films.

Under different ratios of HMPP and HCPK, the gelation yields of UV-cured films prepared using MAEP-PDMS-1 as the prepolymer were inferior to those prepared with DAEP-PDMS-1 as the prepolymer. This was because the content of acryloyloxy functional groups contained in the DAEP-PDMS-1 oligomer was significantly higher than that of the MAEP-PDMS-1 oligomer. In addition, although the gelation yield of UV-cured films prepared using MAEP-PDMS-1 as the prepolymer fluctuated somewhat with the different ratios of HMPP and HCPK, the overall difference was not significant. Based on the cost of the curing agent, the dosage of HMPP and HCPK was still selected as 1:1.

#### 3.2.2. Effect of UV Irradiation Time and Molecular Weight on Curing Performance

**Figure 7:**
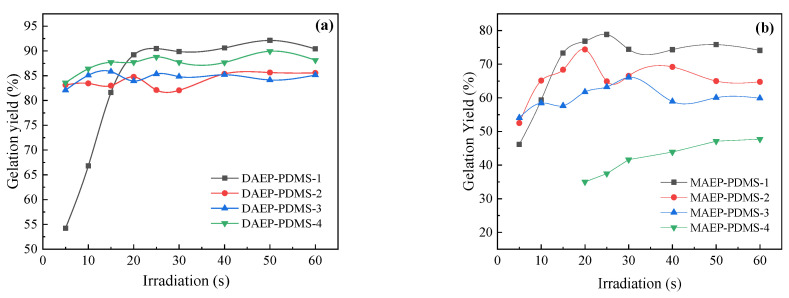
Effect of irradiation time on the gelation yield of cured films ((**a**) with DAEP-PDMS oligomers as the prepolymer; (**b**) with MAEP-PDMS oligomers as the prepolymer).

In order to investigate the effect of the molecular weight of DAEP-PDMS oligomers, that is, the content of reactive acrylate functional groups at both ends of polysiloxane, on the UV curing process and the structure of UV-cured products, the DAEP-PDMS of different molecular weights prepared in Table 1 were used as prepolymers and mixed evenly with the DBABP-PDMS oligomer at a mass ratio of 70:30. After that, a photoinitiator consisted of a mixture of HMPP and HCPK at a mass ratio of 1:1 was added in an amount of 2 wt% of the total mass of the oligomer mixtures. Each component was poured into the mold and irradiated at different times to prepare UV light-curing films according to the method described in Section 2.4. The gelation yield of each cured film was measured according to the method described in Section 2.2, and the results are shown in Figure 7a. When DAEP-PDMS oligomers were substituted by MAEP-PDMS oligomers, mixed evenly with the same DBABP-PDMS oligomer and the hybrid photoinitiator, the obtained mixtures were then irradiated in a drawer-type UV light curing machine for different times to prepare UV-cured films. The gelation yield of each cured sample was measured according to the method described in Section 2.2, and the results are shown in Figure 7b.

It can be seen in Figure 7a that for the UV curing system composed of DAEP-PDMS and DBABP-PDMS, the gelation yield of each UV-cured sample gradually increased with the radiation time when the same initiator composition and dosage was used. After 20 s of UV irradiation, the gelation yield of each sample tended to be stable, and the changes were minor. The strength of the cross-linked network formed by the UV irradiation of acryloyloxy functional groups depended on the content of acryloyloxy functional groups in the mixture and the energy of UV light irradiations the mixture absorbed. Since the power of the drawer-type UV curing machine was fixed, the length of UV irradiation determined the amount of energy absorbed by the UV curing system. It affected the reaction rate and the degree of cross-linking in the UV curing system. For the UV curing system composed of an oligomer with a smaller molecular weight, like DAEP-PDMS-1, the content of acryloxy functional groups was higher than that of other oligomers with a more considerable molecular weight, so a longer irradiation time was required to obtain enough energy to make more acryloxy functional groups cross-linked or vulcanized completely to form a solid network structure. Therefore, the mixture composed of DAEP-PDMS-1 needs to be irradiated for 15 to 20 s to obtain a cured film with a gelation yield of 85%. Among the four oligomers investigated, DAEP-PDMS-4 had the most significant molecular weight, which made the UV curing system composed of it have the lowest acryloxy functional group content. Therefore, the UV light energy required for curing this mixture was the lowest. After irradiation for 10 s, the gelation yield of this mixture reached 86.4%. For the same reason, the gelation yields of two UV curing systems composed of a DBABP-PDMS oligomer and a DAEP-PDMS oligomer with an *M*_NMR_ molecular weight of 3090 g/mol or 2130 g/mol were 85.1% and 83.5%, respectively, after UV irradiation for 10 s. 

For the UV curing system composed of MAEP-PDMS and cross-linking density promoter DBABP-PDMS, the gelation yield of each UV-cured film gradually increased as the irradiation time prolonged. Under the same UV irradiation time, the gelation yield of the UV-cured film showed a downward trend as the molecular weight of the MAEP-PDMS oligomer increased, as shown in Figure 7b. Since there was only one active acryloxy group in the end group of the MAEP-PDMS oligomer, when this acryloxy functional group was conducted with the addition of polymerization with the multiple acryloxy functional groups in the DBABP-PDMS oligomer, the DBABP-PDMS oligomer was end-capped by the MAEP-PDMS oligomer. No macromolecules with dense network structures could be obtained, which resulted in lower gelation yields relative to DAEP-PDMS. In the DAEP-PDMS oligomer, there were two active acryloxy groups in both end groups of the oligomer, and they could participate in the chain growth reactions between the DAEP-PDMS oligomer and the DBABP-PDMS oligomer to build a macromolecule with a dense structure and higher gelation yield. 

#### 3.2.3. FT-IR Spectra of Cured Films 

**Figure 8:**
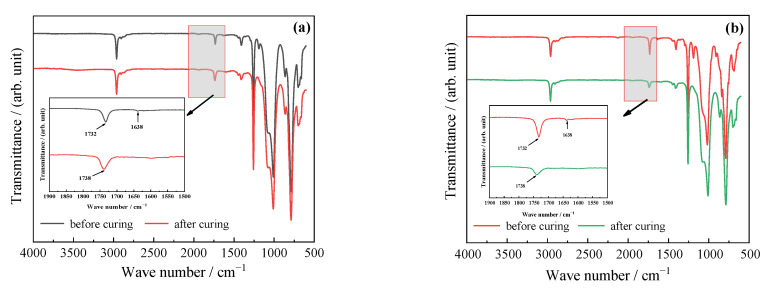
FT-IR spectra of mixtures before and after UV curing ((**a**) with DAEP-PDMS-1 as the prepolymer; (**b**) with MAEP-PDMS as the prepolymer).

When DAEP-PDMS-1, DBABP-PDMS, and the photoinitiator mixture (HMPP and HCPK mixed at a mass ratio of 1:1) were blended evenly at a mass ratio of 70:30:2 and irradiated for 40 s in a drawer-type UV curing machine, a UV-cured silicone film was prepared. The FT-IR spectra of the mixture before and after UV curing are shown in Figure 8a. When DAEP-PDMS-1 was substituted by MAEP-PDMS-1, a UV-cured film was prepared in the same way. The FT-IR spectra of the mixture consisting of MAEP-PDMS-1, DBABP-PDMS, and the mixed photoinitiator before and after UV curing are shown in Figure 8b. It can be seen in Figure 8a,b and their respective enlarged figures between 1900 and 1600 cm^−1^ that the C=C stretching vibration peak at 1638 cm^−1^ disappeared after curing. At the same time, the stretching vibration absorption peak attributed to the C=O group blue-shifted from 1732 cm^−1^ before curing to 1738 cm^−1^ after curing, which resulted from the changes in the chemical environment. The shear vibration of the terminal methylene group attributed to the vinyl group at 1409 cm^−1^ was weakened, indicating the formation of a cross-linked network structure through the addition of polymerization of the C=C bond under the action of free radicals generated by the photoinitiator when the mixture was exposed to UV irradiation. 

#### 3.2.4. Water Absorption Degree of UV-Cured Films

As can be seen in Table 5, when DAEP-PDMS and DBABP-PDMS were used to conduct a free radical addition polymerization under UV irradiation, the degree of reaction (Table 1) in preparing DAEP-PDMS oligomers had a significant influence on the water absorption of the UV-cured films. The DHEP-PDMS oligomer was a hydroxyl-terminated polysiloxane. If the degree of reaction were lower (DAEP-PDMS-2 in Table 1), the resulting DAEP-PDMS oligomer would contain more hydroxyl groups. Since these residual hydroxyl groups were hydrophilic and could not participate in the free radical addition polymerization reaction under UV irradiation, the UV-cured film was not dense. This resulted in a higher water absorption degree. As the degree of condensation reaction increased (Table 1), more acryloxy functional groups that could participate in the UV curing reaction were grafted into the polysiloxane oligomers to form a dense network structure after UV curing and decreased the water absorption degree. In addition, the content of acryloxy functional groups or the number of polysiloxane segments in DAEP-PDMS oligomers was also one of the critical factors affecting the water absorption of UV-cured films.

Although the degree of reaction was low when preparing MAPE-PDMS oligomers, water molecules could not quickly enter the network structure of the cured films because hydrophobic trimethylsilyl functional groups were contained in these oligomers and their precursors, which made the water absorption degrees of all the free radical addition polymerization products of MAPE-PDMS and DBABP-PDMS to be no more than 5%, as shown in Table 5. As the molecular weight of the MAPE-PDMS oligomer increased, the content of UV-curable acryloxy functional groups contained in each copolymer molecule decreased. When the MAPE-PDMS oligomer was copolymerized with DBABP-PDMS, more hydrophobic dimethylsiloxane segments were introduced into the polymer, which decreased the water absorption degree of the UV-cured film with the increase in molecular weight of the MAPE-PDMS oligomer.

#### 3.2.5. Thermal Properties of UV-Cured Films

(1) TGA

**Figure 9:**
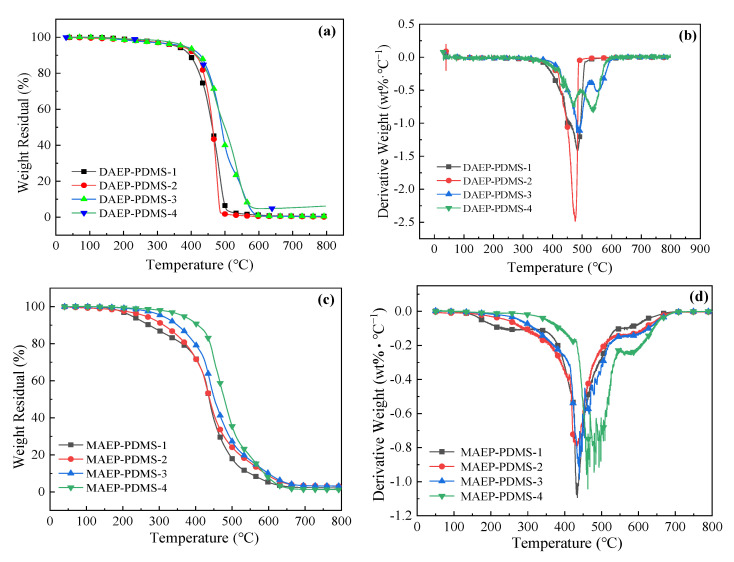
TGA and DTG curves of UV-cured films (DAEP-PDMS as the prepolymer (**a**,**b**); MAEP-PDMS as the prepolymer (**c**,**d**)).

The cured films presented in Figure 7a using DAEP-PDMS as the prepolymer, DBABP-PDMS as the cross-linking density promotor, and irradiated for 40 s were subjected to TGA testing under a nitrogen atmosphere. During the test, the heating rate was 20 °C/min, the airflow rate was 15 mL/min, and the temperature range was 40~800 °C. The obtained TGA curve is shown in Figure 9a, and its corresponding DTG curve is shown in Figure 9b. The temperature at 5% weight loss (*T*_5%_), the temperature at 50% weight loss (*T*_50%_), and the temperature at the maximum thermal degradation rate (*T*_Max_) of the UV-cured film could be determined by the TGA and DTG curves.

It can be seen in Figure 9a,b that the decomposition of four UV-cured films with different molecular weights in nitrogen consisted of three stages. The weight loss in the first stage was less than 10%, and its corresponding temperature ranged from 200 to 350 °C. The weight loss in this temperature range originated from the incompletely cured substrate or easily decomposed organic carbon chains. The UV-cured film underwent a second mass degradation process at temperatures ranging from 400 to 600 °C. The weight loss in this stage mainly came from the decomposition of organic molecules on the repeating unit of PDMS oligomers caused by the breakage of Si-C bonds. The third thermal degradation stage occurred between 450 and 650 °C. Within this temperature range, the thermal degradation behavior of UV-cured films originated from the rearrangement of polysiloxane molecular segments.

It can be seen in Figure 9a that temperatures at a weight loss of 5% (*T*_5%_) for these four UV-cured films that originated from four kinds of DAEP-PDMS oligomers with different molecular weights were 355.9 °C, 368.0 °C, 378.5 °C, and 381.2 °C, respectively. Their temperatures at a weight loss of 50% (*T*_50%_) were 462.4 °C, 463.6 °C, 490.5 °C, and 498.9 °C, respectively. These TGA test data indicated that the DAEP-PDMS oligomers of different molecular weights were rapidly cross-linked after UV irradiation and formed a dense, cross-linked network. UV-cured films not only had a high gelation yield but also had good heat resistance. It can also be found in Figure 9a,b that the thermal properties of each UV-cured film were further enhanced as the molecular weight of the DAEP-PDMS oligomer increased. The reason for this phenomenon might be related to the increase in the number of Si-O-Si segments with higher bond energy as the molecular weights of DAEP-PDMS oligomers increase. 

The cured films presented in Figure 7b using MAEP-PDMS as the prepolymer and DBABP-PDMS as the cross-linking density promotor irradiated for 40 s were subjected to TGA testing under the same conditions. The obtained TGA curves and DTG curves are shown in Figure 9c,d. It can be seen in Figure 9c,d that the thermal degradation temperature of the UV-cured product also increased as the molecular weight of MAEP-PDMS oligomers increased. For mixtures containing MAEP-PDMS oligomers, the thermal degradation process of their UV-cured films in nitrogen also consisted of three stages. The first thermal degradation stage occurred before 150 °C. At this stage, the UV-cured film decomposed slowly. The second thermal degradation process occurred between 150 and 500 °C, and the UV-cured film decomposed rapidly within this temperature range. The third thermal degradation stage occurred between 500 and 800 °C. In this stage, segment chains of the polymer rearranged as they did in the third thermal degradation stage of UV-cured films initiated from DAEP-PDMS oligomers. It can also be seen in Figure 9c,d that compared with the UV-cured films with DAEP-PDMS as the prepolymers, the heat resistance of the UV-cured film with MAEP-PDMS as the prepolymers was obviously inferior to the former. On the other hand, it could also be found from the gelation yield data shown in Figure 7a,b that the gelation yields of the UV-cured films with DAEP-PDMS as the prepolymers were significantly greater than those of the UV-cured films with MAEP-PDMS as the prepolymers, which indicated that the UV-cured film prepared using DAEP-PDMS as prepolymer had a denser network structure and thus exhibited better thermal properties than films prepared from MAEP-PDMS prepolymers.

(2) DSC

**Figure 10:**
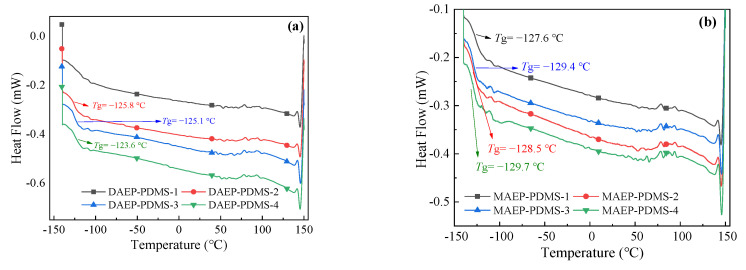
DSC curves of UV-cured films ((**a**) with DAEP-PDMS as the prepolymer; (**b**) with MAEP-PDMS as the prepolymer).

Each UV-cured film irradiated for 40 s, as presented in Figure 7a,b, with DAEP-PDMS or MAEP-PDMS as the prepolymer, was subjected to DSC testing. The obtained DSC curves are shown in Figure 10a,b.

**Figure 11:**
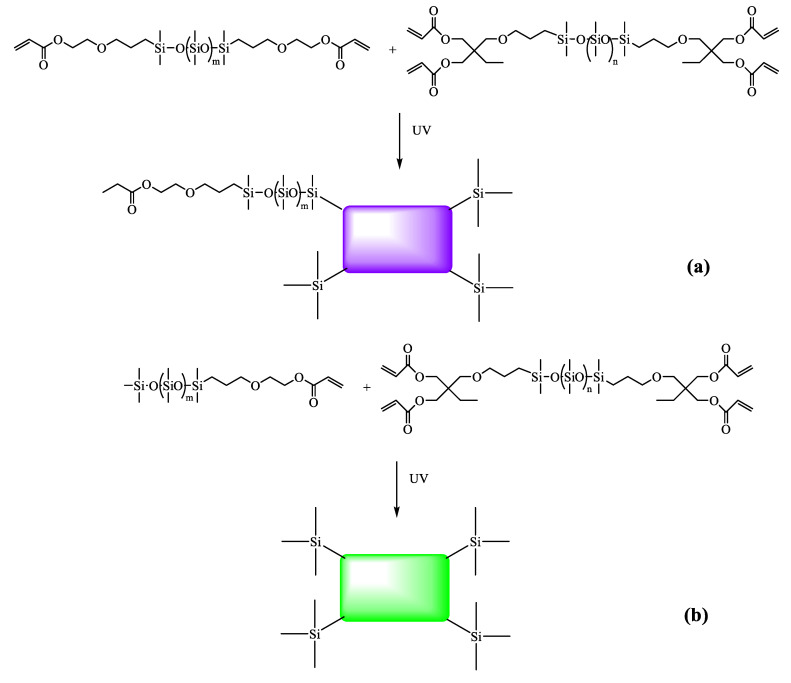
Schematic structural diagram of the presumed copolymer products of the DAEP-PDMS-1 oligomer (**a**) and the MAEP-PDMS-1 oligomer (**b**) with DBABP-PDMS.

As can be seen in Figure 10a, except for the UV-cured film prepared from the mixture of DAEP-PDMS-1 with the smallest molecular weight and DBABP-PDMS, an apparent glass transition temperature was observed for other UV cure films with a higher molecular weight DAEP-PDMS oligomer as the prepolymer in the temperature range of −125.8 °C~−123.6 °C. Moreover, this glass transition temperature range was consistent with the glass transition temperature of polydimethylsiloxane. It can be seen in Figure 7a that the DAEP-PDMS-1 oligomer had the lowest molecular weight and the highest content of acryloxy functional groups. When the DAEP-PDMS oligomer was mixed with the cross-linking density promoter DBABP-PDMS oligomer, the acryloyloxy groups at the terminal positions of the DAEP-PDMS oligomer could interact with other DAEP-PDMS oligomers during the UV curing process to produce polymers with symmetrical structures. Additionally, it could also perform a free radical addition reaction with some of the terminal acryloyloxy group in the DBABP-PDMS oligomer to generate polymers with an asymmetric structure, as shown in Figure 11a. Among the four DAEP-PDMS oligomers examined, the DAEP-PDMS-1 oligomer with the highest content of the acryloxy functional group was more likely to interact with a specific end group of DBABP-PDMS than other DAEP-PDMS oligomers with higher molecular weights to generate a polymer with an asymmetric structure. These asymmetrically structured polymers destroyed the symmetry of the polymer structure and, therefore, eliminated the glass transition temperature, as shown in Figure 10a.

The DSC curves of the UV-cured films prepared using MAEP-PDMS as the prepolymer and mixed with the cross-linking density promotor DBABP-PDMS are shown in Figure 10b. In the temperature range of −129.7 °C~−127.6 °C, apparent glass transition temperatures were observed in the UV-cured films prepared from the four MAEP-PDMS oligomers with different molecular weights, which indicated that all of these UV-cured films had a highly ordered structure. Although each of the MAEP-PDMS oligomers had an asymmetric end-group structure, when they were copolymerized with the cross-link density promotor DBABP-PDMS, the four acryloxy groups in the DBABP-PDMS oligomer could be copolymerized with a terminal acryloxy group in the MAEP-PDMS oligomer to form a trimethylsilyl-terminated polymer with a symmetrical structure, as illustratively shown in Figure 11b. Due to the symmetry in their molecular structures, these polymers exhibited distinct glass transition temperatures. 

#### 3.2.6. Cross-Linking Density of UV-Cured Films

The cross-linking density reflects the tightness or relaxation of the internal network structure of the cured film. The cross-linking density measurement results of UV-cured films prepared by mixing DAEP-PDMS or MAEP-PDMS oligomers with different molecular weights with the cross-linking density promotor DBABP-PDMS at the same mass ratio are shown in Table 6. It can be seen in Table 6 that when each cured film reached swelling equilibrium, the cross-linking density (*v*_e_) of the cured product prepared with DAEP-PDMS as the prepolymer was significantly greater than that of the cured product prepared with MAEP-PDMS as the prepolymer. On the other hand, as the molecular weight of the DAEP-PDMS oligomer increased, the cross-linking density of the UV-cured film showed a decreasing trend. In contrast, the length of the polymer chain segment between two adjacent cross-linking sites gradually increased. Since there was only one curable acryloxy functional group in the MAEP-PDMS oligomer, the cross-linking density of UV-cured films prepared with the MAEP-PDMS oligomer as the prepolymer was significantly lower than that of UV-cured films prepared with the DAEP-PDMS oligomer as the prepolymer. At the same time, the chain segment length between two adjacent cross-linking sites of this kind of UV-cured film was also significantly higher than that of the UV-cured film prepared with the DAEP-PDMS oligomer as the prepolymer, indicating that the cross-linked network structure of the UV-cured film prepared with the DAEP-PDMS oligomer as the prepolymer was relatively relaxed. 

#### 3.2.7. Water Contact Angle of UV-Cured Films

**Figure 12:**
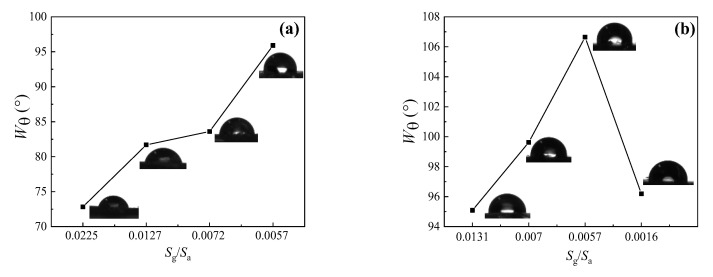
Water contact angle of UV-cured films ((**a**) with DAEP-PDMS as the prepolymer; (**b**) with MAEP-PDMS as the prepolymer; *S*_g_/*S*_a_, a criterion reflecting the relative content of acryloxyl group in the DAEP-PDMS oligomer, which was the ratio of the peak area of one proton attributed to the C=C group in the terminal acryloyloxyethyloxypropyl functional group to the peak area of one proton attributed to the Si-CH_3_ group in the ^1^H NMR spectrum of the oligomer).

The water contact angles of UV-cured films prepared using DAEP-PDMS or MAEP-PDMS oligomers as the prepolymers and DBABP-PDMS as the cross-linking density promoter are shown in Figure 12a,b.

When the DAEP-PDMS oligomer was used as the prepolymer and since there was a polyether segment in the DAEP-PDMS oligomer and the oxygen atom in the polyether structure could form a hydrogen bond with water molecules, the water contact angle of the UV-cured film prepared with a low molecular weight DAEP-PDMS oligomer was less than 90° and showed hydrophilicity. As the molecular weight of the oligomer increased, the ratio between *S*_g_/*S*_a_, a criterion reflecting the relative content of the acryloxyl group in the DAEP-PDMS oligomer, decreased. However, the polysiloxane segment content in the UV-cured films increased, and the water contact angle of the UV-cured films gradually increased from 72.8° to 95.9°, exhibiting a transition from hydrophilic to hydrophilic. In addition, the volume shrinkage that occurred during the UV curing process could lead to an increase in the surface roughness of the cured film. The more acryloxy functional groups are involved in the UV curing process, the rougher the surface produced and the smaller the water contact angle [37].

The water contact angles of each cured were film prepared with MAEP-PDMS, as the prepolymer was greater than 90° and was hydrophobic. As the molecular weight of the MAEP-PDMS oligomer increased, the ratio between *S*_g_/*S*_a_ decreased, indicating that the concentration of the hydrophilic ether group in the oligomer decreased. At this time, the hydrophobic dimethylsiloxane segments contained in the UV-cured film increased, and the water contact angle of the UV-cured films showed an overall increasing trend. Another important reason why the cured films prepared using MAEP-PDMS as the prepolymer were hydrophobic might be related to the unique asymmetric molecular structure of these MAEP-PDMS oligomers. In the MAEP-PDMS oligomer, there was an active acryloxy functional group on one end of the molecule and an inert trimethylsilyl group on the other end of the oligomer. As shown in Figure 11b, during the UV curing reaction process of the mixture composed of MAEP-PDMS and DBABP-PDMS, the acryloxy groups in the MAEP-PDMS oligomers and the acryloxy groups in the DBABP-PDMS oligomers copolymerized by means of a free radical addition polymerization mechanism, and the inert trimethylsilyl-terminated polysiloxane segments were grafted onto the DBABP-PDMS molecules, causing the resulting UV-cured film to be hydrophobic.

Due to the lower degree of reaction in preparing the MAEP-PDMS-4 copolymer (Table 3), the hydroxyl groups in the MAEP-PDMS-4 oligomer failed to react fully with AC. Therefore, there were more hydrophilic hydroxyl groups remaining in the MAEP-PDMS-4 oligomer, which led to an unexpected decrease in the water contact angle of the UV-cured film prepared using the MAEP-PDMS-4 oligomer as the prepolymer. 

## 4. Conclusions

A series of DAEP-PDMS, MAEP-PDMS, and DBABP-PDMS oligomers with different molecular weights were prepared through the condensation reaction between the hydroxyl group located at the end group of the polysiloxane oligomer and AC. Structures of the obtained oligomers were carefully characterized using NMR, FT-IR, and GPC. The reaction degree of acryloyloxy functional polysiloxane prepared by the condensation reaction of hydroxyl functionalized polysiloxane and AC was between 67.4% and 85.1%. As the molecular weight of the oligomer increased, the reaction degree generally exhibited a downward trend. 

When the photoinitiator HMPP and HCPK were mixed at a mass ratio of 1:1, and the photoinitiator dosage was 2 wt% of the total mass of the prepolymer and cross-linking density promotor, the gelation yield of the UV-cured film reached the maximum value after 20 s of UV irradiation. When the DAEP-PDMS-1 oligomer was used, the gelation yield of the UV-cured film was 89.2%. When the MAEP-PDMS-1 oligomer was used, the gelation yield of the UV-cured film was 85.8%. 

The gelation yield of the UV-cured films could be effectively enhanced when oligomers with multiple UV-curable acryloxy functional groups were blended with linear PDMS terminated by either two acryloxy groups at each end of the oligomer or just one acryloxy group at one end of the oligomer. When DAEP-PDMS or MAEP-PDMS oligomers with different molecular weights were used as critical components of the UV curing system, the gelation yield of the UV-cured films exhibited a trend of increasing rapidly at first and then gradually became steady as the UV irradiation time increased. When DAEP-PDMS was used as the main component of the UV curing system, the gelation yields of the UV-cured films obtained by irradiation for 20 s were greater than 85%. The content of acryloxy functional groups contained in the molecules of the DAEP-PDMS oligomer decreased with the increase in the molecular weight of DAEP-PDMS oligomers, and the water absorption degree of the cured film formed by free radical addition polymerization of the DAEP-PDMS oligomer and the DBABP-PDMS oligomer exhibited an overall downward trend. However, the thermal properties of UV-cured films formed from DAEP-PDMS oligomers and DBABP-PDMS improved as the molecular weight of the oligomers increased. When the MAEP-PDMS oligomer was used as the main component of the UV curing system, the gelation yield of the UV-cured films was lower than that of the UV-cured films obtained using the DAEP-PDMS oligomer with a similar molecular weight and irradiation for the same periods. As the molecular weight of the MAEP-PDMS oligomer increased, the content of acryloyloxy functional groups contained in the MAEP-PDMS oligomer decreased.

Consequently, the water absorption degree of the cured film formed by free radical addition polymerization of the MAEP-PDMS oligomer and DBABP-PDMS oligomer showed an overall downward trend. Compared with the polymer network formed by the DAEP-PDMS oligomer, the water absorption degree of the polymer network formed by the MAEP-PDMS oligomer was lower, which might be directly related to the particular trimethylsilyl-terminated end group structure in the MAEP-PDMS oligomer. Although the thermal degradation temperature of UV-cured films formed by MAEP-PDMS oligomers also increased with the increase in molecular weight, these thermal degradation temperatures were much lower than those of UV-cured films using DAEP-PDMS oligomers with similar molecular weights.

Except for the DAEP-PDMS-1 oligomer with a lower molecular weight, apparent glass transition temperatures were observed in the UV-cured films formed by the DAEP-PDMS oligomers with a more considerable molecular weight. Apparent glass transition temperatures were observed in the UV-cured films formed by one of the four MAEP-PDMS oligomers investigated in this manuscript. As the molecular weight of the DAEP-PDMS oligomer increased, the cross-linking density of the UV-cured films formed by DAEP-PDMS oligomers decreased, and the average chain segment length between two adjacent cross-linking sites showed a gradually increasing trend. The cross-linking density and the average chain segment length between two adjacent cross-link sites of the UV-cured films formed using MAEP-PDMS instead of DAEP-PDMS showed a similar trend. However, the value of the cross-linking density of the UV-cured films was lower than DAEP-PDMS oligomers with similar molecular weights.

The water contact angle value of the cured film formed by the copolymerization of DAEP-PDMS or MAEP-PDMS and DBABP-PDMS showed a gradually increasing trend as the molecular weight of the oligomer increased. For DAEP-PDMS oligomers, when its *M*_NMR_ molecular weight gradually increased from 1180 g/mol to 4150 g/mol, the water contact angle of the UV-cured films formed by the copolymerization of the DAEP-PDMS oligomer and DBABP-PDMS gradually increased from 72.8° to 95.9°, indicating a surface property transition of the cured films from hydrophilic to hydrophobic. When the MAEP-PDMS oligomer was used, the water contact angles of the cured films formed by copolymerization with DBABP-PDMS were greater than 90° and showed hydrophobicity. As the molecular weight of the oligomer increased, the water contact angle of the UV-cured film showed a gradually increasing trend.

## Figures and Tables

**Table 1:** Synthesis of DAEP-PDMS oligomers with different molecular weights.

Entry	*M*n ^a^ (g·mol^−1^)	*m* ^b^ _DHEP-PDMS_ (g)	*m* ^c^ _AC_ (g)	*m* ^d^ _TEA_ (g)	*m* ^e^ _DCM_ (g)	*m* ^f^ _DAEP-PDMS_ (g)	*M*n ^g^ (g·mol^−1^)	Reaction Degree (%)	Yield (%)	PDI	*η*_25_ (cP)
DAEP-PDMS-1	1050	30.00	5.18	5.79	30.00	26.28	1180	84.7	74.7	1.90	32.75
DAEP-PDMS-2	1910	30.00	2.84	3.18	30.00	23.03	2130	78.1	70.1	2.17	31.50
DAEP-PDMS-3	3020	30.00	1.80	2.01	30.00	24.21	3090	93.7	76.1	1.97	73.25
DAEP-PDMS-4	4010	30.00	1.35	1.51	30.00	23.26	4150	82.4	74.2	2.12	95.00

Note: ^a^. The molecular weight of DHEP-PDMS, calculated by ^1^H NMR spectra; ^b^. The mass of DHEP-PDMS; ^c^. The mass of acryloyl chloride; ^d^. The mass of triethylamine, ^e^. The mass of dichloromethane; ^f^. The mass of triethylamine; ^g^. The molecular weight of DAEP-PDMS, calculated by ^1^H NMR spectra.

**Table 2:** Experimental results for preparing MAMP-PDMS oligomers.

Entry	Precursor	*M* ^a^ _NMR_ (g·mol^−1^)	*m* ^b^ (g)	*m* ^c^ _AHE_ (g)	*m* ^d^ _Cat._ (g)	*m* ^e^ (g)	Yield (%)
MAMP-PDMS-1	MDMS-PDMS-1	550	20.00	3.74	0.24	21.58	90.90
MAMP-PDMS-2	MDMS-PDMS-2	960	30.00	3.17	0.33	30.93	93.25
MAMP-PDMS-3	MDMS-PDMS-3	1440	18.86	1.34	0.20	18.23	90.27
MAMP-PDMS-4	MDMS-PDMS-4	2220	30.00	1.38	0.31	29.30	93.37

Note: ^a^. The molecular weight of the MDMS-PDMS oligomer, determined by ^1^H NMR spectra; ^b^. The mass of the MDMS-PDMS oligomer; ^c^. The mass of the allyl hydroxyethyl ether (AHE); ^d^. The mass of Karstedt’s catalyst with a Pt content of 0.1 wt%; ^e^. The mass of the MAMP-PDMS oligomer.

**Table 3:** Experimental results for preparing MAEP-PDMS oligomers.

Entry	Intermediate	*m* ^a^ (g)	*M*_NMR_ (g·mol^−1^)	*m*_AC_ (g)	*m*_TEA_ (g)	*m*_DCM_ (g)	*m* ^b^ (g)	Reaction Degree (%)	Yield (%)	*η*_25_ (cP)
MAEP-PDMS-1	MAMP-PDMS-1	15.00	650	2.09	2.34	30.00	14.55	89.5	85.14	7.25
MAEP-PDMS-2	MAMP-PDMS-2	15.00	1060	1.28	1.42	30.00	12.54	86.8	77.03	15.75
MAEP-PDMS-3	MAMP-PDMS-3	18.00	1550	1.06	1.21	36.00	13.78	72.8	72.30	64.00
MAEP-PDMS-4	MAMP-PDMS-4	15.00	2220	0.61	0.68	30.00	10.73	39.0	68.74	41.75

Note: ^a^. The mass of the MAMP-PDMS oligomer; ^b^. The mass of the MAEP-PDMS oligomer.

**Table 4:** Effect of the photoinitiator on the gelation yield of UV-cured films with DAEP-PDMS-1 or MAEP-PDMS-1 as a prepolymer.

Entry	Prepolymer	*m*_HMPP_/*m*_HCPK_	Gelation Yield (%)
A1	DAEP-PDMS-1	1:0	89.1
A2	DAEP-PDMS-1	1:1	89.2
A3	DAEP-PDMS-1	2:1	89.3
A4	DAEP-PDMS-1	3:1	89.3
A5	DAEP-PDMS-1	4:1	89.8
A6	DAEP-PDMS-1	5:1	89.5
B1	MAEP-PDMS-1	1:0	81.0
B2	MAEP-PDMS-1	1:1	85.8
B3	MAEP-PDMS-1	2:1	81.6
B4	MAEP-PDMS-1	3:1	82.8
B5	MAEP-PDMS-1	4:1	85.4
B6	MAEP-PDMS-1	5:1	81.2

Note: The ratio between the total mass of HMPP and HCPK and the total mass of DAEP-PDMS-1 (or MAEP-PDMS-1) and DBABP-PDMS was 2%; DAEP-PDMS-1 in Table 1 (or MAEP-PDMS-1 in Table 3) was blended with the DBABP-PDMS at a mass ratio of 70: 30, and the UV curing time was 20 s.

**Table 5:** Water absorption results of UV-cured films imitated from DAEP-PDMS or MAEP-PDMS oligomers.

Entry	*S*_g_/*S*_a_	First Test			Second Test			Third Test			Water Absorption (%)
		*m*_a1_ (mg)	*m*_b1_ (mg)	*m*_c1_ (mg)	*m*_a2_ (mg)	*m*_b2_ (mg)	*m*_c2_ (mg)	*m*_a3_ (mg)	*m*_b3_ (mg)	*m*_c3_ (mg)	
DAEP-PDMS-1	2.25 × 10^−2^	16.9	15.3	26.7	17.2	15.6	15.6	16.8	14.8	26.0	5.65
DAEP-PDMS-2	1.27 × 10^−2^	27.0	40.4	23.0	29.0	44.2	44.2	26.1	39.6	22.6	12.01
DAEP-PDMS-3	0.72 × 10^−2^	37.0	35.4	50.9	36.9	35.8	35.8	35.7	34.8	49.5	3.26
DAEP-PDMS-4	0.57 × 10^−2^	26.3	23.3	50.0	26.1	25.5	25.5	25.7	23.4	47.8	4.57
MAEP-PDMS-1	1.31 × 10^−2^	46.7	46.9	44.5	27.6	26.3	25.0	15.3	14.7	14.3	4.15
MAEP-PDMS-2	0.70 × 10^−2^	32.8	33.0	31.4	19.5	19.9	18.9	31.0	31.6	30.4	4.63
MAEP-PDMS-3	0.57 × 10^−2^	35.1	35.2	34.2	22.9	23.1	22.7	64.9	63.8	62.9	2.00
MAEP-PDMS-4	0.16 × 10^−2^	47.7	47.7	45.9	27.3	26.5	26.2	23.0	22.5	22.2	2.06

Note: *m*_a1_, *m*_a2_, and *m*_a3_ were the initial mass of the sample in each water absorption test; *m*_b1_, *m*_b2_, and *m*_b3_ were the mass of the sample after it was saturated with water in each test; *m*_c1_, *m*_c2_, and *m*_c3_ were the mass of the sample after drying in an oven at 80 °C for 1 h in each test; Sg/Sa represented that in the ^1^H NMR spectrum of each DAEP-PDMS oligomer, the ratio of the peak area of one proton attributed to the C=C group in the terminal acryloyloxyethyloxypropyl functional group to the peak area of one proton attributed to the Si-CH_3_ group.

**Table 6:** Results of the cross-linking density of UV-cured films.

Entry	*ρ* ^a^ (g·cm^−3^)	*W*_0_ ^b^ (g)	*W*_s_ ^c^ (g)	*φ* ^d^ (%)	*v*_e_ ^e^ × 10^−4^ (mol·cm^−3^)	*M*c ^f^ (g·mol^−1^)
DAEP-PDMS-1	0.67	0.11	0.16	0.75	39.3	172
DAEP-PDMS-2	0.88	0.11	0.21	0.52	10.0	881
DAEP-PDMS-3	0.89	0.11	0.25	0.44	6.3	1413
DAEP-PDMS-4	0.74	0.10	0.21	0.51	9.7	765
MAEP-PDMS-1	1.00	0.10	0.20	0.45	6.5	1537
MAEP-PDMS-2	0.85	0.13	0.31	0.44	6.0	1412
MAEP-PDMS-3	0.97	0.11	0.25	0.43	5.6	1731
MAEP-PDMS-4	1.11	0.09	0.17	0.48	8.2	1350

Note: ^a^. The density of cured film; ^b^. The initial mass of UV-cured film before being immersed in toluene; ^c^. The mass of UV-cured film reaching swelling equilibrium; ^d^. The volume fraction of polysiloxane in the swelled sample; ^e^. The cross-linking density; ^f^. The average molecular weight of the chain segment between two adjacent cross-linking sites.

## Data Availability

Data are contained within the article and Supplementary Materials.

## Supplementary Materials

The following supporting information can be downloaded at: https://www.mdpi.com/article/10.3390/polym16040465/s1, Figure S1: ^1^H NMR spectrum of a DAEP-PDMS-2 oligomer; Figure S2: ^1^H NMR spectrum of a DAEP-PDMS-3 oligomer; Figure S3: ^1^H NMR spectrum of a DAEP-PDMS-4 oligomer; Figure S4: ^1^H NMR spectrum of an MAEP-PDMS-2 oligomer; Figure S5: ^1^H NMR spectrum of an MAEP-PDMS-3 oligomer; Figure S6: ^1^H NMR spectrum of an MAEP-PDMS-4 oligomer.
