# Supplementary material for: The Preparation of Acryloxyl Group Functionalized Siloxane Polymers and the Study of Their Ultra Violet Curing Properties
Source: Polymers (Basel). 2024 Feb 7;16(4):465. doi: 10.3390/polym16040465 (PMC10893288; doi:10.3390/polym16040465)
Supplement: Supplementary file 1 [file polymers-16-00465-s001.zip › polymers-2771987-supplementary.pdf]

Supplementary Material: The Preparation of Acryloxyl Group Functionalized Siloxane Polymers and the Study of Their Ultra Violet Curing Properties

Dan Du, Xupeng Chen, Yue Wu, Chuan Wu, Zhirong Qu, Yanjiang Song, Dawei Qin, Qiao Li and Hong Dong

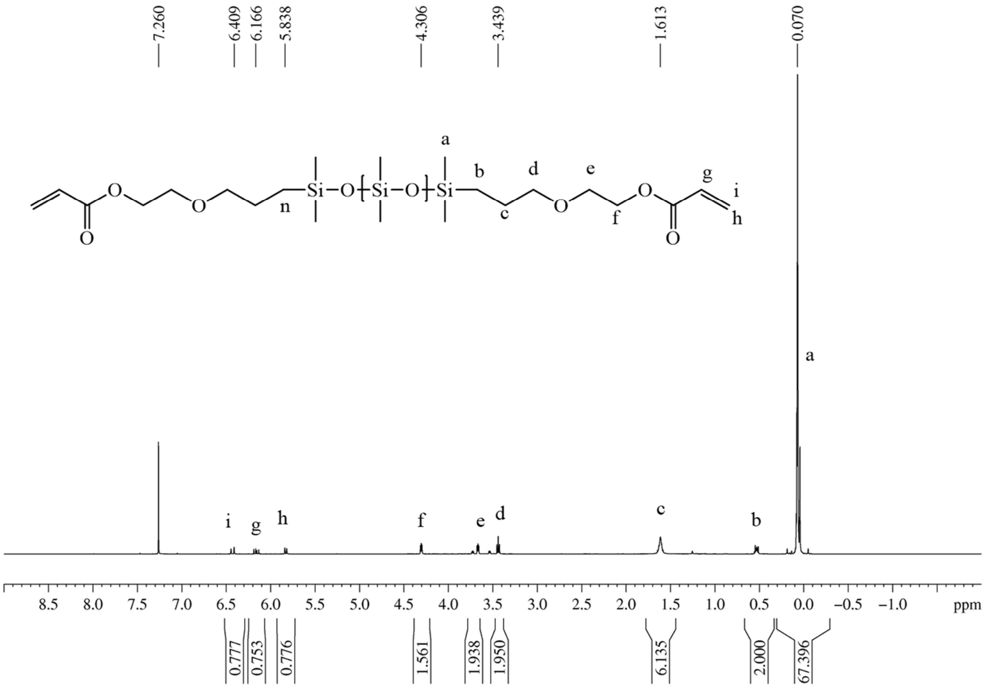

Figure S1. ^{1}H NMR spectrum of DAEP-PDMS-2 oligomer.

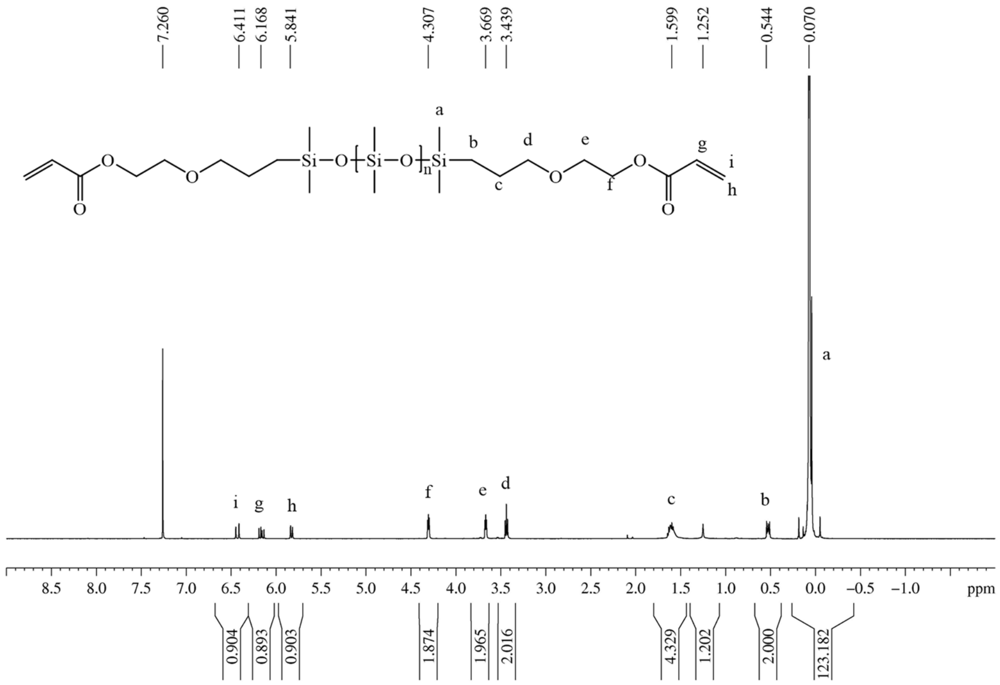

Figure S2. ^{1}H NMR spectrum of DAEP-PDMS-3 oligomer.

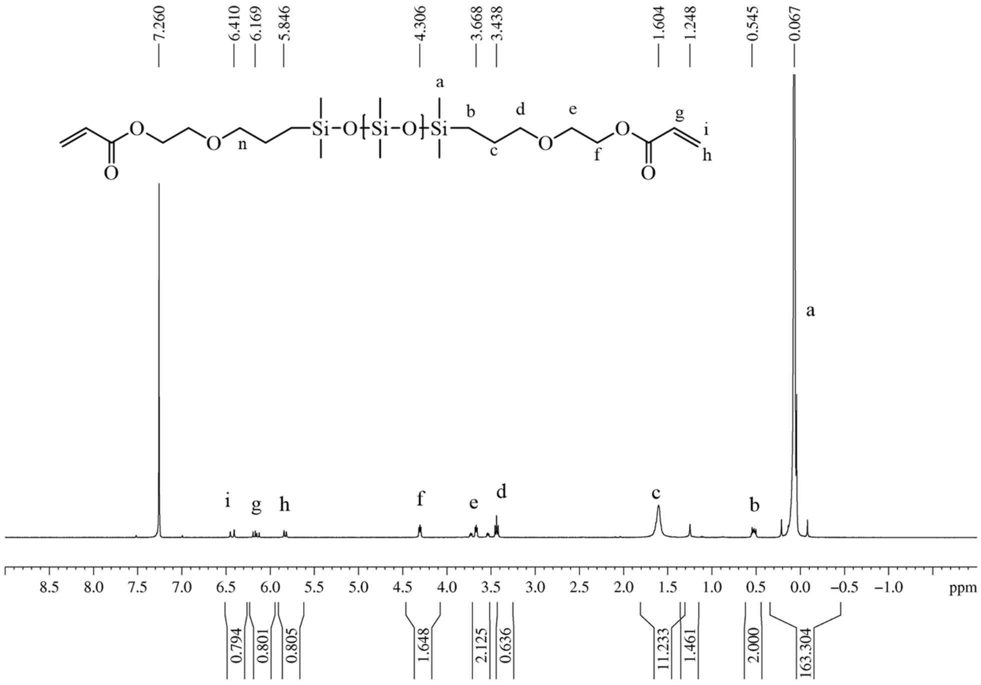

Figure S3. ^{1}H NMR spectrum of DAEP-PDMS-4 oligomer.

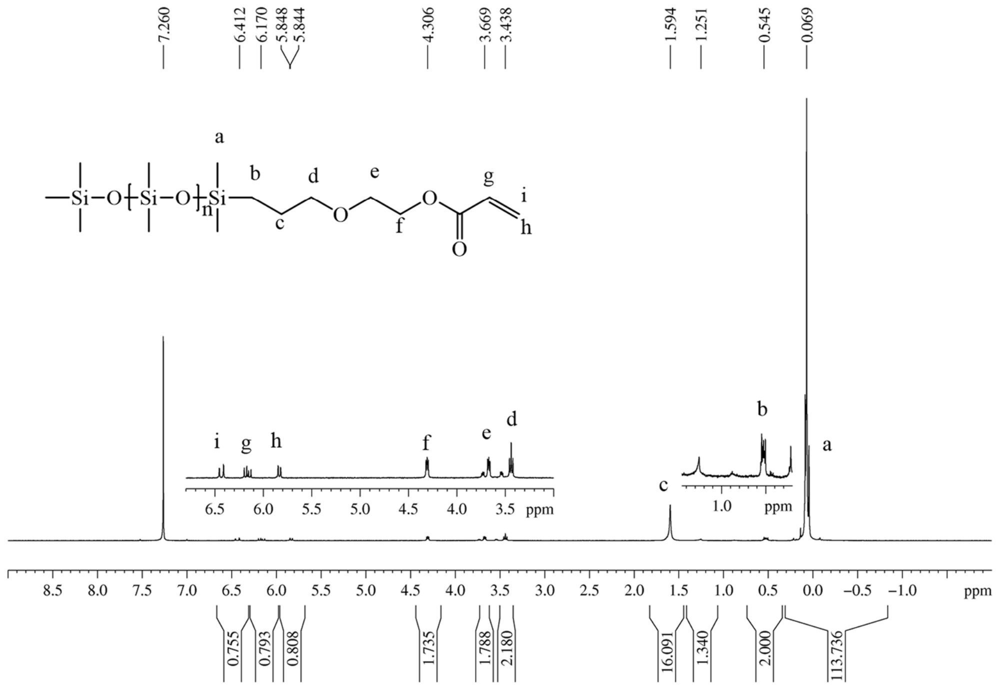

Figure S4. ^{1}H NMR spectrum of MAEP-PDMS-2 oligomer.

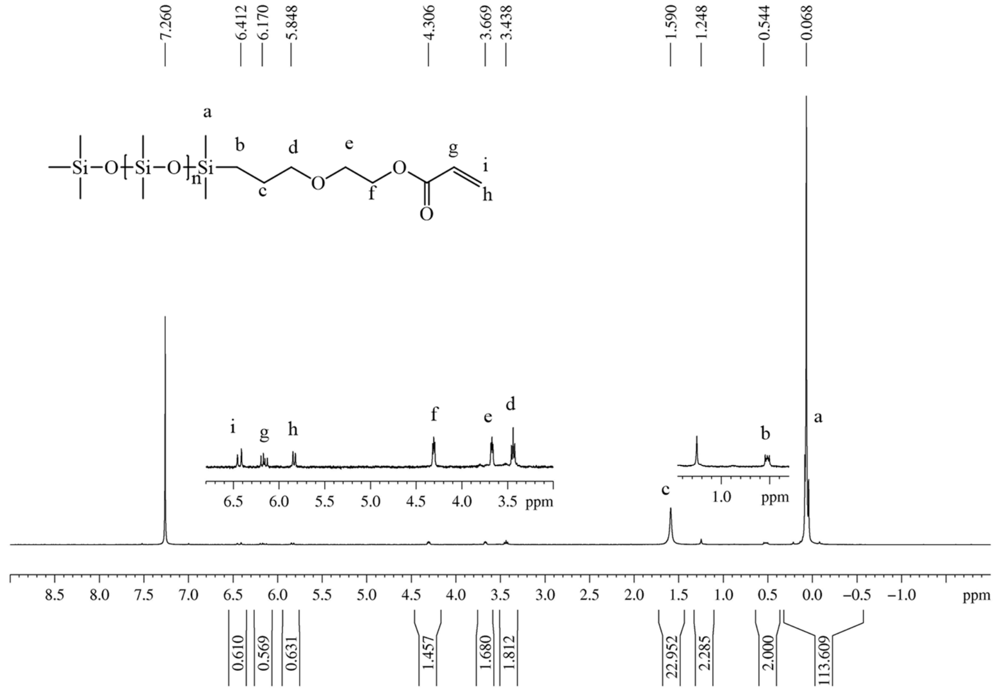

Figure S5. ^{1}H NMR spectrum of MAEP-PDMS-3 oligomer.

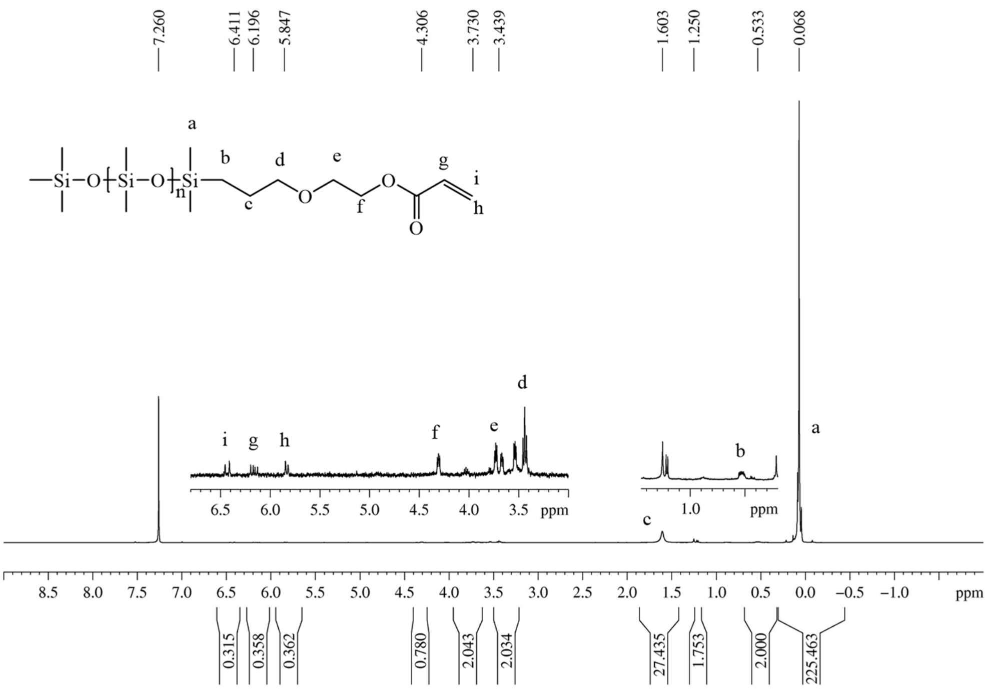

Figure S6. ^{1}H NMR spectrum of MAEP-PDMS-4 oligomer.
